# Symbiotic bacteria-mediated imbalance and repair of immune homeostasis: exploring novel mechanisms of microbiome-host interactions in atopic dermatitis

**DOI:** 10.3389/fimmu.2025.1649857

**Published:** 2025-07-23

**Authors:** Xingyue Lai, Jilin Huang, Yulin Li, Liang Dong

**Affiliations:** School of Medical and Life Sciences, Chengdu University of Traditional Chinese Medicine, Chengdu, Sichuan, China

**Keywords:** atopic dermatitis (AD), skin microbiome, symbiotic bacteria, immune, autoimmune skin disease

## Abstract

The skin surface is colonised by a rich microbiome, and intricate interactions between this microenvironment and microbial communities are critical for maintaining skin homeostasis. Atopic dermatitis (AD), a chronic inflammatory skin disease characterised by skin barrier dysfunction and aberrant immune activation, exhibits a rising global incidence. While conventional therapeutic strategies offer short-term symptom control, their long-term use is limited by adverse effects including skin atrophy, metabolic disorders, and increased infection risk. Critically, these approaches fail to cure AD or reverse the underlying immune imbalance. Recent research has firmly established the skin microbiome as a central driver in AD pathogenesis. The molecular mechanisms underpinning microbiome-host interactions, including the potential for remote regulation via the gut-skin axis, are now being actively investigated. This review systematically analyses how microbial dysbiosis in AD promotes Th2/Th17 immune polarization through three key pathways: microbial metabolites, immune signalling, and barrier integrity. Building on these mechanistic insights and recent advances, we propose novel multimodal therapeutic strategies targeting the microbial-immune axis. We further elucidate the role of commensal bacteria in maintaining immune homeostasis. Ultimately, this synthesis aims to bridge fundamental research with clinical applications, providing a robust theoretical foundation for future therapeutic development and clinical studies in AD management.

## 1 Introduction

As the largest organ and primary defense barrier, the skin’s complex anatomical structure and heterogeneous microenvironment create a unique microbial ecosystem (1). A bidirectional Gut-Skin Axis (GSA) links these organs through microbial metabolites, immune signals, and environmental factors (2). Both systems utilize tightly linked proteins (e.g., claudins) to maintain physical barriers and synergistically defend against pathogens. Existing studies have shown that commensal flora such as *Staphylococcus epidermidis(S. epidermidis)* can secrete novel antimicrobial peptides to inhibit *Staphylococcus aureus (S. aureus)* colonisation and regulate IL-1β expression in keratinocytes via the A20 protein, allowing for the fine-tuning of immune homeostasis (3).

AD is a chronic inflammatory disease driven by skin barrier dysfunction and aberrant immune activation, affects up to 20% of children and 3% of adults globally with rising incidence (4, 5). Its hallmark symptoms—intense itching, dryness, erythema, and exudative lesions—stem directly from epidermal permeability barrier disruption (6, 7). This complex pathology involves genetic susceptibility (e.g., *FLG* mutations), environmental triggers, and immune dysregulation.

Current clinical management of AD encompasses topical anti-inflammatory agents (glucocorticoids, TCS; calcineurin inhibitors, TCIs), systemic immunosuppressants (e.g., cyclosporine, methotrexate) for moderate-to-severe cases, and monoclonal antibody biologics such as dupilumab (anti-IL-4Rα) that inhibit the Th2 pathway (8). Although these therapies provide symptomatic control in AD, prolonged use of topical corticosteroids (TCS) causes adverse effects including skin atrophy, telangiectasia, and metabolic disturbances. Concurrently, systemic immunosuppressive agents may induce hepatorenal toxicity and increase infection susceptibility (9), which highlight the need for exploring new pathogenic mechanisms and targeting them for the necessity of therapeutic treatment. Notably, probiotic-based microbial transplantation has recently demonstrated efficacy in restoring flora balance and alleviating AD symptoms, marking a pivotal transition from mechanistic research to clinical translation in skin microbiome science (10).

## 2 Symbiotic bacteria on the skin surface

Human skin acts as a physical barrier to prevent the entry of pathogenic microorganisms while providing a home for commensal bacteria and fungi, and functional studies have demonstrated the impact of specific strains on modulating the immune system, shaping the microbial community, providing colonisation resistance and promoting epidermal barrier integrity (11). Recent studies have integrated the microbiome, immunity and tissue integrity to understand their interactions in common diseases such as AD.

### 2.1 Composition and function

The bacterial diversity of the skin microbiome is dominated by *Actinobacteria*, *Firmicutes* and *Proteobacteria*, with *S. aureus* and *S. epidermidis* occupy significant ecological niches on the skin surface. Multi-omics analyses revealed that *S. aureus*, while exhibiting low abundance on healthy skin, was substantially enriched in lesional skin of AD patients. Critically, its absolute abundance demonstrated a strong positive correlation with disease severity (12). In contrast, plasma coagulase-negative *S.epidermidis*, as a symbiotic bacterium, forms biofilms by secreting polysaccharide intercellular adhesins (PIA), inhibits pathogen colonization (13), and reduces the degree of inflammation by regulating host TLR3 signaling pathway (14). Similarly, *Staphylococcus hominis (S. hominis)* is also a bacterium with negative plasma coagulase. *S. hominis* can secrete autoinducing peptides to inhibit the expression of harmful protease EcpAd and prevent the increase of pathogenic bacteria (15). Similarly, antimicrobial peptides (AMPs) derived from microorganisms, particularly short peptide bacteriocins (SPBs) and quorum sensing inhibitory peptides (AIPs) produced by symbiotic bacteria, hold significant therapeutic potential in AD treatment by selectively inhibiting the growth and virulence of *S. aureus*, modulating skin immune responses, and restoring skin microbiota balance (16). *Cutibacterium acnes (C. acnes* formerly *Propionibacterium acnes*) predominates in sebaceous gland-rich regions, where it metabolizes sebum triglycerides to produce short-chain fatty acids (SCFAs). These SCFAs maintain skin acidity and enhance barrier function by suppressing inflammatory factor release (17). Conversely, *CERS1*—a molecular biomarker uniquely correlated with *S. aureus* abundance—may drive skin barrier dysfunction through fatty acid sequestration. This represents a maladaptive compensatory response to reductions in very long-chain fatty acids, *ELOVL6* expression, and short-chain sphingolipid composition (18). In contrast, cutaneous fungi—predominantly *Malassezia* species—exhibit high dependency on host-derived lipids for colonization. *Malassezia globosa*, which lacks fatty acid synthase (FAS) genes, relies on lipase-mediated hydrolysis of sebum for nutrient acquisition. This metabolic adaptation drives its niche-specific enrichment in sebaceous-rich regions (19). Notably, these findings align with recent experimental evidence demonstrating that exogenous lipids ameliorate AD pathology in murine models by rectifying immune dysregulation and microbiota imbalances (20). Conversely, the virulent phage group dominates this niche, with its abundance dynamically linked to host bacterial community structure (21).

### 2.2 Spatial distribution

**Figure 1:**
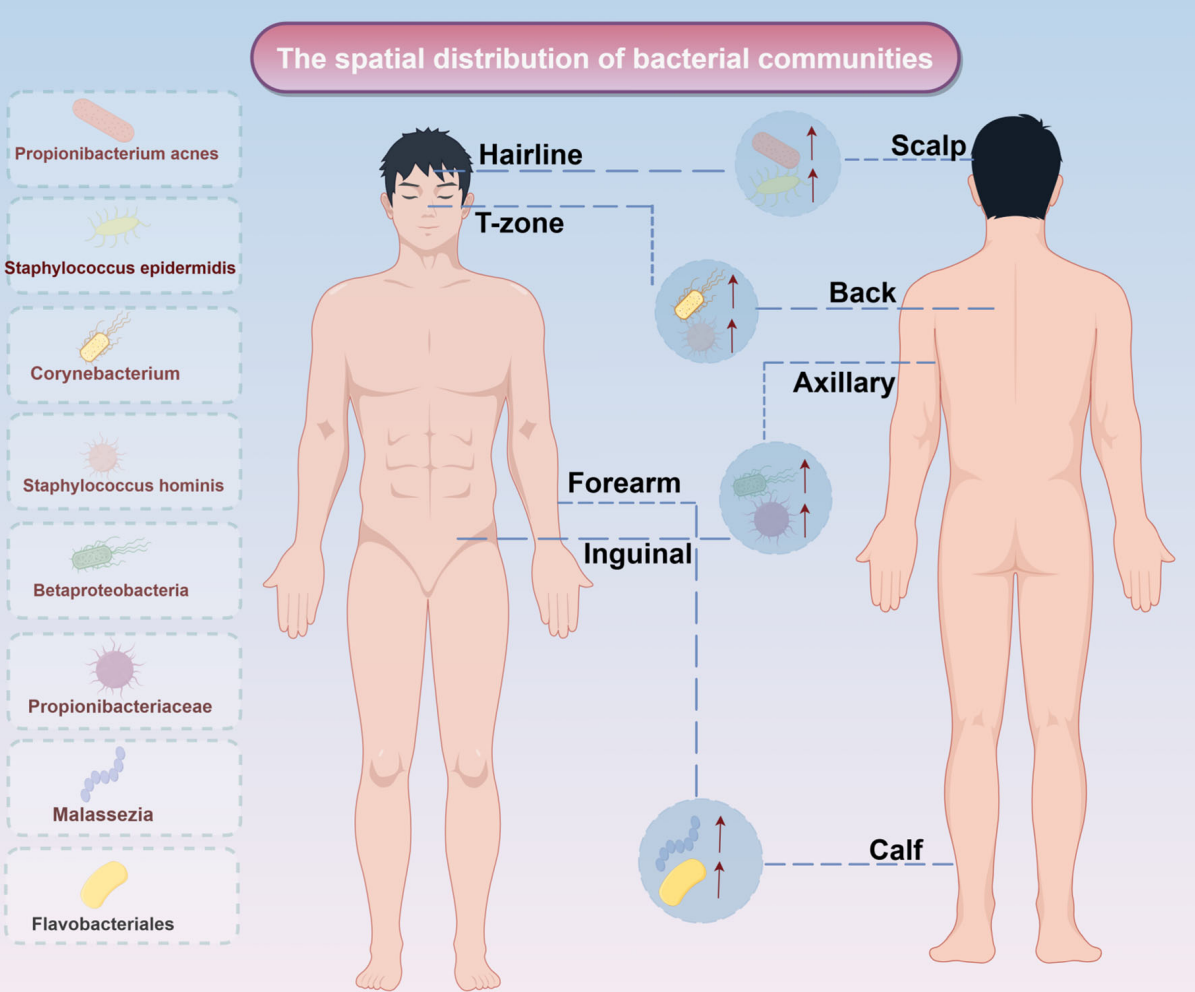
Spatial distribution of bacterial communities. The spatial distribution of bacterial communities in different parts of the human body. The left side displays the main colonized bacterial genera or groups, including Propionibacterium acnes and Staphylococcus epidermidis; the right side corresponds to specific areas of the human body (such as the hairline, T-zone, scalp, etc.), reflecting the spatial heterogeneity of microbial communities on the skin and body surface.↑: increase expresion.

The skin microbiota exhibits significant spatial heterogeneity driven by local physicochemical properties. Lipophilic species, including *C. acnes* and *S. epidermidis*, dominate sebum-rich zones (T-zone, back), where they hydrolyze sebum triglycerides to modulate barrier function. However, overgrowth of these species can trigger inflammatory pathologies like acne (22). In contrast, moist intertriginous areas (axillae, groin) feature high eccrine gland density and humidity, shaping distinct microbial communities (23). Due to the high density of sweat glands and high humidity, *Corynebacterium* and *S. hominis* are often enriched in humid environments, in which *Corynebacterium* produces volatile thiols through the metabolism of branched-chain amino acids in sweat, which are involved in the formation of body odour, and the dry areas such as the forearms and the calves are characterised by the proliferation of β-Aspergillus phylum (e.g., Betaproteobacteria), which are also involved in the regulation of the skin barrier function (24). Betaproteobacteria and Flavobacteriales exhibit low abundance but high diversity in skin regions exposed to external environments, a pattern linked to frequent epidermal desquamation. Meanwhile, specialized niches like the scalp and hairline harbor unique microbial communities dominated by Propionibacteriaceae and *Malassezia* fungi, reflecting distinct follicular structures and sebum secretion dynamics (25) (**Figure 1**).

Skin microenvironmental factors—temperature, humidity, pH, and lipid content—function as ecological filters that modulate microbial community assembly (26). High humidity promotes the colonization of *S. epidermidis*, and the ceramides secreted by it enhance the water retention ability of skin barrier, forming a mutually beneficial network between “bacteria and host” (27). However, a high PH environment is conducive to the survival of *S. aureus* (28). Sebum-derived free fatty acids exhibit dual functionality: they possess antimicrobial properties while serving as a carbon source for *C. acnes*. This bacterium further regulates cutaneous pH through secretion of SCFAs, establishing a closed-loop equilibrium (29).

The skin microbiome in AD patients exhibits characteristic dysbiosis. Lesional areas show significant enrichment of *S. aureus*, which secretes δ-toxin to disrupt keratinocyte tight junctions (30). Concurrently, depletion of commensal *S. epidermidis* and *C. acnes* impairs anti-inflammatory SCFAs synthesis (31). AD patients exhibit reduced diversity of Betaproteobacteria in non-lesioned dry skin, indicating systemic immune dysregulation exerts distal effects on microbiome composition. Notably, skin creases in AD show inverted *Corynebacterium*-to-*Staphylococcus* ratios. This dysbiosis correlates with impaired antimicrobial peptide secretion driven by localized overexpression of Th2 cytokines IL-4 and IL-13 (32).

## 3 The role of commensal bacteria in immune homeostasis

Commensal bacteria critically establish mucosal immune tolerance by regulating immune cell differentiation and cytokine networks. Polysaccharide A (PSA) from *Bacteroides fragilis* drives CD4+ T cell differentiation into *Foxp3+* Treg cells and induces anti-inflammatory cytokine secretion (e.g., IL-10) (33). These effects occur via Toll-like receptor 2 (TLR2) signaling. This process enhances Treg immunosuppressive capacity and alleviates inflammation in experimental colitis; symbiotic metabolites thereby actively regulate immune tolerance. IL-10 directly inhibits the antigen-presenting function of dendritic cells (DCs), whereas TGF-β promotes the differentiation of peripheral Tregs by inducing the expression of *Foxp3*.TGF-β induces peripheral initial T cells into Treg with immunosuppressive function, a process that significantly reduces Th2-type inflammatory responses in oral immunotherapy of AD patients. Microbial-derived SCFAs regulate Treg/Th17 balance through epigenetic and receptor-mediated pathways. SCFAs such as butyrate and propionate inhibit histone deacetylase (HDAC), enhancing histone acetylation in the *Foxp3* promoter region to promote Treg differentiation (34). SCFAs inhibit mTOR-S6K signaling via GPR43 activation, blocking RORγt-mediated Th17 differentiation. This bidirectional regulation occurs in both gut and skin. Intestinal commensal bacteria-derived ATP promotes Th17 differentiation through CD11c+ dendritic cells (DCs), while SCFAs counteract this process to maintain immune homeostasis. Separately, commensal bacterial flagellin activates TLR5 on DCs, inducing IL-6 secretion that modulates Th17 differentiation thresholds. These mechanisms reveal precise microbial ligand-immune cell interactions.

*S. epidermidis* can upregulate the expression of *FLG*, and indirectly produce natural moisturizing factor (NMF) to enhance the compactness of the stratum corneum, so as to enhance the skin barrier function (35). Cutaneous symbiotic bacteria produce Indole-3-acetic acid (IAA), indole-3-acetaldehyde and indolepyruvate through tryptophan metabolism, which enhance epithelial barrier function via aryl hydrocarbon receptor (AHR) signaling pathway. This study lays the foundation for the development of skin disease therapy based on microbial metabolism (36). Clinically, loss-of-function *FLG* mutations in AD patients cause barrier defects that facilitate *S. aureus* colonization and promote Th2-type inflammation (37, 38). Commensal bacteria directly inhibit pathogen proliferation through antimicrobial peptide secretion (e.g., lantibiotics like lugdunin). Separately, S*. epidermidis*-derived phenol-soluble modulins (PSMs) disrupt *S. aureus* biofilms and attenuate virulence. This ‘ecosystem competition’ mechanism is critically important during AD’s acute phase, driving pathogen dominance (39). Microbiome-targeted therapies show significant potential for AD management.

## 4 Immune characteristics and immune imbalance in AD

AD is an immune disease, and its occurrence usually involves the driving of some immune factors and the imbalance of immune mechanisms.

### 4.1 AD immune characteristics and core driving factors

AD is an autoimmune skin disease, which is usually associated with some immune mechanisms. The following will be described from three aspects:

#### 4.1.1 Th2-driven immune dysregulation

The immune profile of AD is characterized by predominant Th2 differentiation of naïve CD4^+^ and CD8^+^ T cells. These Th2 cells secrete IL-4, IL-5, and IL-13—core drivers of the inflammatory cascade (40). Upon binding to keratinocyte IL-4Rα/IL-13Rα1 receptor complexes, these cytokines activate the *JAK1/TYK2/JAK2-STAT6/STAT3* pathway. This signaling significantly suppresses filaggrin (FLG) and antimicrobial peptide (AMP) expression, ultimately causing barrier dysfunction (41). IL-4 significantly downregulates key epidermal structural proteins (filaggrin, loricrin, involucrin) and keratin-related genes, while simultaneously promoting B-cell class switching to IgE to amplify allergic responses. Notably, IL-13—secreted predominantly in chronic phases—stimulates eosinophilic infiltration and upregulates chemokines CCL17 and CCL22 (42). Dupilumab, a monoclonal antibody targeting IL-4Rα, represents a therapeutic breakthrough in Th2 pathway inhibition. By blocking shared IL-4/IL-13 signaling, it significantly reduces Scoring Atopic Dermatitis (SCORAD) indices and serum IgE levels in AD patients (43). In the AD model of mice with IL-13 gene defects, the activation of Th2 cells is enhanced, and the relative levels of short-chain sphingomyelin (SM) and ceramide (CER), which are composed of non-hydroxy fatty acids and sphingolipids, increase, while the relative levels of long-chain types decrease (44).

#### 4.1.2 Contribution of Th17 to AD heterogeneity

Despite Th2 predominance in AD, Th17 cells contribute significantly to specific AD subtypes (45–47). IL-17A exacerbates epidermal hyperplasia (acanthosis) and neutrophil infiltration by suppressing E-cadherin expression and upregulating S100A proteins (S100A7/8/9). In chronic lesions, Th17-Th2 crosstalk occurs: IL-4 attenuates inflammation through Th17 differentiation inhibition, while Th17-derived IL-22 further compromises barrier function, establishing a pathogenic feedback loop (48). Notably, Asian AD patients exhibit elevated IL-17C expression correlating with epidermal thickening and psoriasiform features. Single-cell sequencing confirms Th1/Th17/Th22 co-infiltration in chronic lesions, indicating spatiotemporal immune dynamics as a key source of disease heterogeneity (49).

#### 4.1.3 Role of aryl hydroxyl receptor in AD

The AHR critically regulates terminal epidermal differentiation.*Ahr*- keratinocytes exhibit significantly reduced expression of key differentiation markers—including FLG, loricrin (LOR), and involucrin (IVL)—impairing stratum corneum formation. Concurrently, AHR deficiency alters cytokine homeostasis: levels of AD-associated cytokines (IL-33, IL-36γ, TSLP) are diminished, while the pro-inflammatory factor IL-24 is elevated. This dysregulation suggests AHR is essential for maintaining cutaneous immune homeostasis (50). Therapeutically, coal tar upregulates filaggrin (FLG) expression in keratinocytes and inhibits STAT6 activation via the AHR/NRF2 axis. This pathway antagonizes IL-4/IL-13-mediated degradation of barrier structural proteins, ultimately ameliorating AD-associated barrier defects (51). The tryptophan (Trp) metabolic pathway is significantly impaired in the skin microbiota of AD patients, resulting in markedly reduced levels of indole-3-carbinol (IAId)—a key microbially derived metabolite. Normally, IAId functions as an endogenous AHR ligand that suppresses thymic stromal lymphopoietin (TSLP) expression via promoter binding, thereby attenuating Th2 inflammation. In AD lesional skin, however, IAId deficiency permits dysregulated TSLP overexpression (52).

### 4.2 Immune dysregulation in AD

The core of the immune imbalance in AD lies in the overactivation of the Th2-type immune response. Research indicates that cytokines IL-4 and IL-13 are key factors driving AD inflammation. These cytokines enhance the STAT6 signaling pathway by activating the IL-4Rα receptor, which promotes the differentiation of Th2 cells and inhibits the expression of filaggrin, a barrier protein in keratinocytes, leading to the disruption of the skin barrier function (53). Meanwhile, in patients with AD, the balance between Th1 and Th2 cells is disrupted. During the acute phase, the immune response is primarily driven by Th2 cells, characterized by high levels of IL-4, IL-5, and IL-13. In contrast, during the chronic phase, the levels of Th1-related factors IFN-γ and TNF-α increase (54). Additionally, IL-17A and IL-22, secreted by Th17 cells, play a dual role in AD, contributing to both antimicrobial defense and potentially exacerbating inflammation (55).

**Figure 2:**
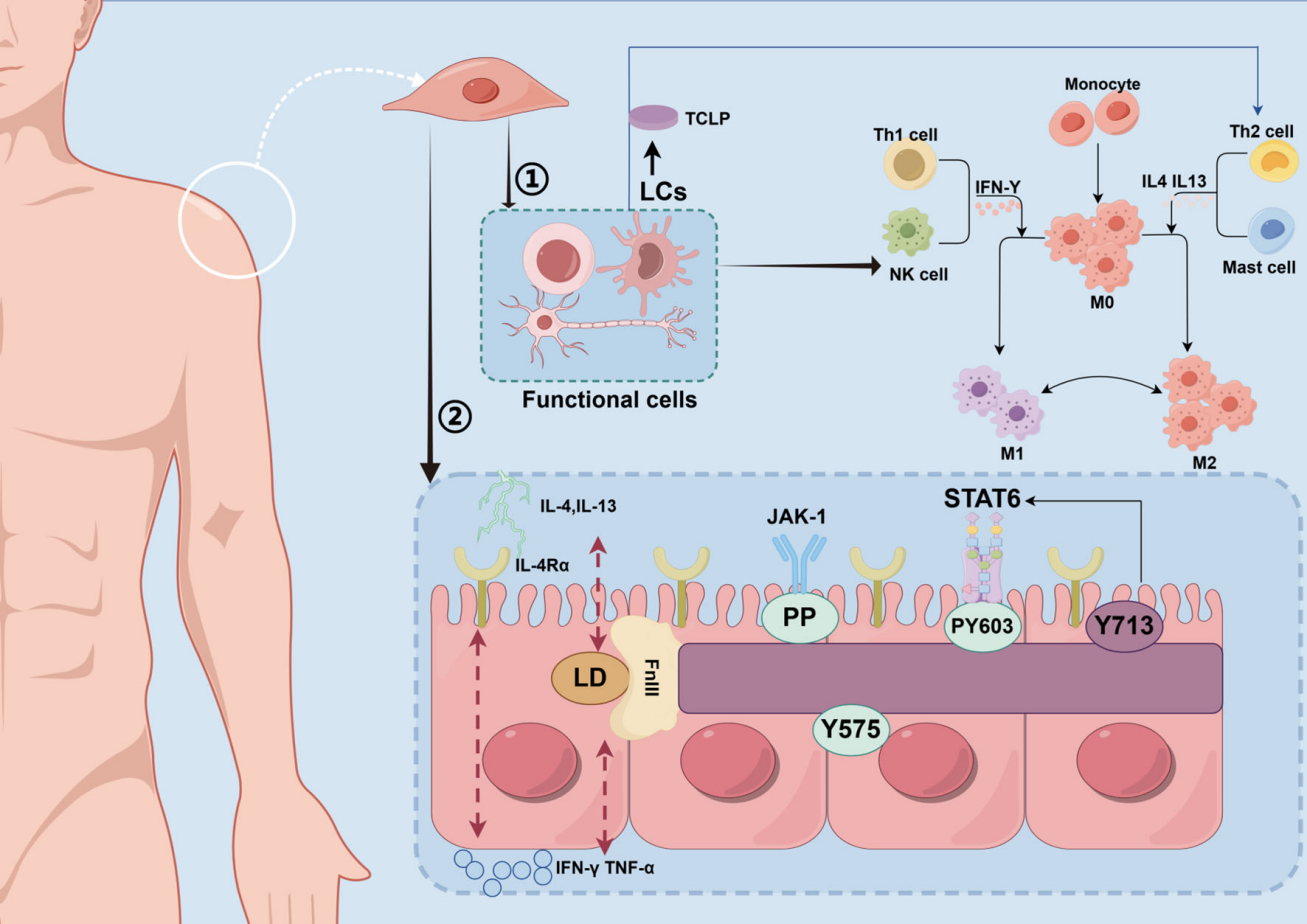
Molecular mechanism of regulating skin immune homeostasis. This figure depicts the molecular mechanisms by which immune cells on the skin surface and commensal microbiota regulate immune homeostasis through the STAT6 signaling pathway. TH2 cells secrete IL-4 and IL-13, which bind to the IL-4 receptor (IL-4Ra) on monocytes and mast cells, activating the JAK-1/STAT6 pathway. Meanwhile, the commensal microbiota may influence STAT6 phosphorylation through metabolites or direct signaling. (LCs, Langerhans cells; TSLP, Thymic Stromal Lymphopoietin; Th1 cell, T Helper 1 Cell; NK cell, Natural Killer cell; IFN-γ, Interferon-gamma; M0, resting macrophages; M1, M1 Macrophages; M2, M2 Macrophages; IL-4, Interleukin-4; IL-13, Interleukin-13; Th2 cell, T Helper 2 Cell; IL-4Rα, Interleukin-4 Receptor Alpha; LD, Linker Domain; FnIII, Fibronectin Type III Domain; TNF-α, Tumor Necrosis Factor Alpha; PP, Phosphorylation; JAK-1, Janus Kinase 1; STAT6, protein; Y575/PY60/Y713, phosphorylation site).

Antigen-presenting cells, such as Langerhans cells (LCs) and dendritic cells (DCs) in the epidermis, play a central role in initiating the AD immune response. These cells take up and present antigens, recruit other immune cells, and regulate the direction of the immune response (56). Recent studies have shown that skin surface symbiotic bacteria (e.g., *S. aureus*) can directly activate DCs and LCs through pattern recognition receptors, promote antigen presentation and T cell differentiation, and lead to the occurrence of AD (57, 58). DCs are crucial for the differentiation of Th2 cells. TSLP, released by damaged keratinocytes, can activate LCs/DCs. Activated DCs then express co-stimulatory molecules like OX40L, which drive the differentiation of initial T cells towards Th2 (59). Additionally, experiments have shown that IL-33 activates DCs through a MyD88-dependent signaling pathway, promoting the secretion of Th2-type cytokines, a mechanism confirmed in AD mouse models (60). In addition, inflammatory dendritic epidermal cells (IDECs) are also involved in the pathogenesis of AD. IDECs express high levels of FcϵRI receptor, which binds to IgE-allergen complex and releases pro-inflammatory mediators such as TNF-α and IL-12, thus amplifying Th1 and Th17 responses (**Figure 2**).

## 5 The vicious cycle of “microflora imbalance-immune activation-barrier destruction” in AD

In AD, skin microbiome dysbiosis and immune dysregulation form a self-reinforcing pathological loop. This immune deviation impairs barrier function by suppressing filaggrin (FLG) and antimicrobial peptide (AMP) synthesis, establishing a ‘dysbiosis–immune activation–barrier disruption’ cycle.

### 5.1 Bacterial imbalance in AD

Analysis of lesional skin microbiomes in AD patients revealed significant dysbiosis. Commensal bacteria—including *Streptococcus*, *Cutibacterium*, and *Corynebacterium*—showed reduced abundance. In contrast, *S. aureus* colonized >90% of lesions, with its abundance positively correlating with disease severity (61, 62). This dysbiosis impairs production of antimicrobial peptides (LL-37, β-defensin) while altering the skin microenvironment through bacterial metabolites. Both mechanisms promote *S. aureus* adhesion and growth. Reduced microbiome diversity strongly correlates with AD relapse. During acute episodes, the number of *S. aureus* increased significantly. After treatment, inflammation subsided as symbiotic bacteria such as *streptococcus* and *S. epidermidis* recovered (63). Choi et al. observed increased proportions of *L. fermentum* strain SLAM216 in the gut and identified LF216EV. In mice, LF216EV elevated *Limosilactobacillus* and *Lactococcus* abundance while alleviating AD symptoms. This therapeutic effect may involve altered expression of serotonin-related genes (*htr2c*, *sert*, *tph1*) (64).

The skin microbiome of AD patients differs significantly from that of healthy individuals. Multicentre clinical studies reveal a substantial reduction in microbial diversity within AD lesional skin. Shannon’s index decreases by 40–60% in these regions. Concurrently, *S. aureus* dominates, reaching relative abundances of 70–90% (65). For example, a 16S rRNA sequencing cohort study of Indonesian AD patients revealed distinct microbial shifts. In moderate AD skin, Firmicutes constituted up to 85% of the microbiome. *S. aureus* abundance increased 8-fold compared to healthy controls. In contrast, mild AD skin showed Proteobacteria predominance (60%). This study also documented, for the first time, the presence of the commensal bacterium *Ensifer adhaerens *(66).

### 5.2 Immune activation mediated by dysbiosis in AD

**Figure 3:**
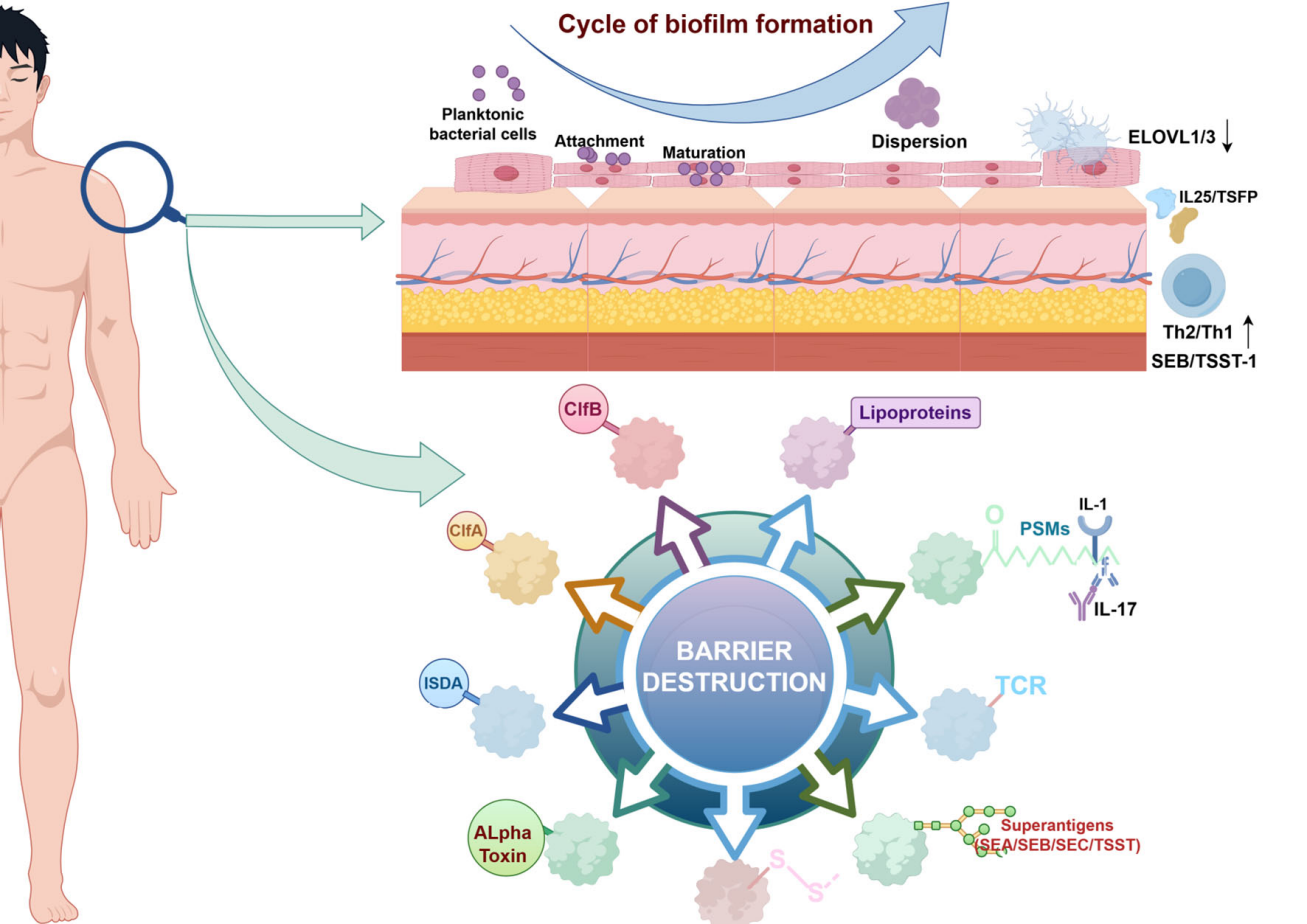
Mechanisms of skin barrier damage and biofilm formation. *S aureus* has highly evolved multiple cell-wall proteins and secreted factors that enable adhesion to human skin and barrier disturbance by using physical, chemical, and inflammatory mechanisms. Adhesion,*S aureus* has developed several surface molecules to adhere to the human stratum corneum, including clumping factors A and B (ClfA and ClfB). Barrier destruction, *S aureus* a-toxin, a water-soluble cytotoxin, forms a heptameric b-barrel pore in host cell membranes. In the epidermis it directly forms pores in keratinocytes, which erodes the integrity of the epidermal barrier. *S aureus* produces at least 10 proteases, a number of which facilitate dissolution of the stratum corneum. In addition to secreted proteases, *S aureus* can directly stimulate endogenous keratinocyte proteases, highlighting an additional mechanism toward barrier destruction. Proinflammatory mechanisms, Cell-wall bound protein A, when solubilized, triggers inflammatory responses from keratinocytes through TNF receptor (TNFR). Staphylococcal superantigens, such as SEA, SEB, SEC, and toxic shock syndrome toxin-1 (TSST-1), trigger B-cell expansion and cytokine release. *S aureus* secretes PSMs, which are direct proinflammatory drivers with compartment-specific effects. In the epidermal compartment PSMs stimulate IL-36a–driven gd T cell– mediated inflammation, whereas in the dermal compartment they stimulate IL-1b–driven Th17 inflammation. (↓, decrease expression; ↑, increase expression; ELOVL 1/3, Elongation of Very Long chain fatty acids1/3; IL 1/17/25, Interleukin 1/17/25 ;TSFP, Thymic Stromal Folliculin Protein; Th 1/2, Th1/2 cell, T Helper 1/2 Cell; SEA/SEB/SEC, Staphylococcal Enterotoxin A/B/C; TSST-1, Toxic Shock Syndrome Toxin-1; ISDA, *Staphylococcus aureus* protein; ClfA/B, Clumping Factor A/B; PSMs, Peptide-Spectrum Matches; TCR, T cell receptor; -S-S-, Disulfide Bond.

Microbiome dysregulation reconfigures the skin immune landscape via multiple pathways. *S. aureus* overgrowth directly activates keratinocytes through δ-toxin secretion, triggering TSLP and IL-33 release to drive Th2-polarized immunity (67). *S. aureus* enterotoxins SEB and TSST-1 function as superantigens that bind MHC class II molecules and T-cell receptor (TCR) Vβ regions independent of antigen presentation. This activates polyclonal T cells, triggering massive release of IL-31 (directly inducing pruritus) and IFN-γ (driving chronic inflammation). Clinically, elevated serum IgE against these superantigens in AD patients correlates significantly with eczema area severity index (EASI) scores (68). Beyond bacterial dysbiosis, fungal community disruption exacerbates skin inflammation—particularly in head/neck AD—by activating pattern-recognition receptors (PRRs) to drive pathogenic IL-17 secretion (69) (**Figure 3**).

Moreover, AD microbiota frequently show impaired utilization of 2’-fucosyllactose (2’-FL), indicating disrupted retinol metabolism and consequent immune tolerance defects (70). *S. epidermidis* balances pro- and anti-inflammatory responses by inducing IL-1β/IL-6 secretion to promote Th17 polarization while upregulating *Foxp3* to enhance regulatory T cell (Treg) function (71, 72). This precise immunomodulatory network operates through microbial metabolite-mediated HDAC inhibition, thereby enhancing anti-microbial peptide (AMP) expression (10, 73).

### 5.3 AD skin barrier disruption and the vicious cycle of immune-microbiome

The normal skin barrier comprises a multilayered defense system: the stratum corneum, granular layer (stratum granulosum), spinous layer (stratum spinosum), and basal layer (stratum basale). The stratum corneum—the core physical barrier—maintains structural integrity through three key components: cross-linked cornified envelope proteins, lamellar lipid bilayers, and NMF (74). Skin barrier dysfunction manifests through two core abnormalities: reduced ceramide content with altered lipid composition in the stratum corneum, and defective intercellular junctions due to downregulated claudin-1 expression. These alterations collectively elevate risks of allergen penetration and microbial colonization (75, 76). In AD patients, diminished microbial diversity coincides with dysbiotic expansion of *S. aureus*. This pathogen forms biofilms that exacerbate skin barrier defects through a synergistic cycle of colonization and inflammation. Targeted microbiome modulation can restore protective flora abundance, including commensals like *Corynebacterium* species that reinforce barrier integrity (77). The protein encoded by the silk protein gene is not only involved in the terminal differentiation of keratinocytes, but its degradation products also regulate the pH of the cuticle and maintain water balance. The tight junction protein Claudin-1 forms a ‘molecular zipper’ structure in the spinous layer to limit molecular permeation (78). In addition, antimicrobial peptides (AMPs) such as β-defensins and cathelicidins form a chemical barrier that enables immunosurveillance by directly killing pathogens and regulating dendritic cell activity.

Approximately 50% of AD patients carry a *FLG* gene Loss-of-function (LOF) mutation that leads to reduced NMF synthesis, dehydrated stratum corneum and increased pH (79), a defect that inhibits keratinocyte differentiation through disturbances in calcium ion signalling. Recent studies have shown that Claudin-1 expression is significantly reduced in AD non-lesional skin, leading to enlargement of tight junction pores and making it easier for allergens to penetrate the epidermis. A 40-60% reduction in stratum corneum ceramides disrupts lamellar body secretion and lipid bilayer organization. This structural defect correlates with impaired peroxisome proliferator-activated receptor gamma (PPARγ) signaling, which normally upregulates lipid synthesis genes. PPARγ also modulates immune polarization, promoting Th2 cells, type 2 innate lymphoid cells (ILC2s), and M2 macrophages that drive inflammation (80, 81). Elevated kallikrein-related peptidase 5/7 (KLK5/7) activity accelerates degradation of corneodesmosomal core proteins (e.g., corneodesmosin), triggering premature stratum corneum desquamation. This pathological process is amplified by downregulated expression of lympho-epithelial Kazal-type inhibitor (LEKTI), a key metalloproteinase inhibitor that normally constrains KLK protease activity (82). Upon barrier disruption, V8 protease (SspA) secreted by *S. aureus* enzymatically cleaves keratin 16 (K16), exposing cryptic antigenic epitopes that trigger pathological immune responses (83).

Notably, the stratified lipid structure of the stratum corneum not only restricts the invasion of pathogenic bacteria through physical isolation, but its low pH environment also maintains commensal dominance by inhibiting the protease activity of *S. aureus*. Upon barrier compromise, downregulation of fatty acid synthesis genes ELOVL1/3 disrupts lipid metabolism, while simultaneously released alarmins (IL-25, TSLP) promote Th2 immune responses. This initiates a self-perpetuating cycle of barrier disruption - microbial dysbiosis - immune dysregulation (84–86). This pathogenic cascade is particularly pronounced in AD. *S. aureus* biofilms disrupt keratinocyte intercellular junctions via α-toxin, while its superantigen SEB binds HLA-DR molecules to activate Vβ T-cell receptor clonal expansion. This induces IL-31-mediated pruritus and drives IL-17/IL-22-dependent chronic inflammation (87). Recent studies demonstrate that specific probiotics (e.g., *Limosilactobacillus reuteri* DYNDL22M62) mitigate AD inflammation through dual mechanisms: restoration of skin microbial diversity; suppression of TSLP production, reducing Th2 cytokine levels. This reverses Th2 immune polarization and provides a molecular foundation for microbiome-targeted therapies (73).

## 6 Clinical applications and future therapies

### 6.1 Traditional treatment

Among JAK inhibitors, abrocitinib showed rapid itch relief in Chinese adults with AD without treatment-emergent serious adverse cardiovascular events (88). JAK inhibitors demonstrate dual mechanistic and clinical efficacy. Baricitinib inhibited JAK-STAT signaling in CD4+ T cells, significantly reducing MAPK and PI3K/Akt/mTOR pathway activity. This achieved a mean 62% SCORAD reduction in 124 Chinese AD patients, confirming its therapeutic utility in Asian populations (89). Topical ruxolitinib cream precisely targets cutaneous JAK1/JAK2 activity, with systemic exposure at 1/1000th of oral administration levels (90). Dupilumab, an IL-4Rα-targeted therapy, demonstrates significant long-term efficacy and safety in moderate-to-severe AD. Real-world evidence confirms sustained EASI improvement >70% at 4 years without significantly elevating infection risk (91). Despite widespread dupilumab use in moderate-to-severe AD, studies indicate increased cutaneous T-cell lymphoma (CTCL) risk (RR = 4.10; 95% CI: 2.06-8.19) (92).

### 6.2 Symbiotic bacteria for the treatment of AD

#### 6.2.1 Treatment based on the gut-skin axis

In randomized controlled trials, oral *Limosilactobacillus fermentum* supplementation reduced SCORAD scores by 40.4% (mean) in pediatric AD patients, with efficacy positively correlating with treatment duration (*p = 0.003*) (93). Notably, *Bifidobacterium* modulates the gut-skin axis by increasing fecal butyrate concentrations. Specific strain combinations (e.g., *Bifidobacterium* CECT 8145 + CECT 7347) restored the *Faecalibacterium*/*Bacteroides* ratio in AD patients’ intestinal flora, providing a molecular rationale for individualized microbial therapies (94).

Clinical trials validate microbiome modulation efficacy via the skin-gut axis. A *Lactobacillus*-based probiotic formulation reduced SCORAD by 34% in pediatric AD, increasing SCFAs while inhibiting Th2 cytokines (95). FMT from healthy donors decreased *S. aureus* skin colonization by 68% in Phase II trials, with IL-31 levels correlating with itch severity (96). *Faecalibacterium prausnitzii* metabolites restored intestinal barrier integrity, lowering plasma LPS and ameliorating skin inflammation (97).

#### 6.2.2 Treatment based on skin prebiotics

Similarly, recent studies have shown that some potential skin prebiotics have therapeutic effects on AD. Professor Richard Gallo’s team identified specific human *S. epidermidis* strains that produce potent antimicrobial peptides that selectively kill *S. aureus* and prevent or alleviate AD symptoms in mouse models (98). *S. hominis A9* has completed Phase I clinical trials, confirming its safety in humans. The small molecule substance <10 kDa secreted by *S. epidermidis* can significantly induce the expression of human β-defensins hBD2 and hBD3 by activating the TLR2 receptor of keratinocytes, thus enhancing the antibacterial ability of skin to *S. aureus*, providing a new idea for the treatment of AD (99). *Roseomonas mucosa* significantly improved symptoms in children with AD, reduced *S. aureus* colonization and repaired the skin barrier through a lipid-mediated TNFR2-EMT repair pathway, and was safe (100). The autologous epidermal *S. hominis A9* strain isolated from the skin of healthy people can secrete antimicrobial peptides (lantibiotics) to directly kill *S. aureus*, and produce autologous induced peptides (AIP) to inhibit the quorum sensing system of *S. aureus*, reduce the expression of toxins (such as PSMα), and alleviate the symptoms of AD (101). Numerous clinical trials have investigated topical probiotics for treating AD, suggesting their potential for widespread clinical application in the future.

### 6.3 Future therapies

Several emerging microbiome-targeted therapeutic strategies are being used.The colonization density of *S. aureus* in the skin of AD patients is increased, and its virulence factors can aggravate skin inflammation, while endolysin can be specifically targeted at *S. aureus.* The study found that the use of endolysin to treat AD can significantly reduce the frequency of AD attacks (102). Antimicrobial peptides (AMPs) restore skin microbiota balance, restore barrier function and reduce inflammation in patients with AD by selectively inhibiting the growth of *S. aureus*, blocking the expression of virulence factors, and regulating skin immune response. In the future, AMPs can be used to treat AD (16). The use of local bacteriophages can reduce the inflammatory indicators of skin diseases, such as the expression of chemokine CXCL2, neutrophil infiltration and other inflammatory cytokines. It may become a new therapy for AD (103).

Future AD therapeutics will focus on multimodal synergistic interventions. Key skin microbiome-targeted approaches include: personalized microbial transplantation and microbial metabolite preparations (e.g., SCFAs). These restore microbial diversity and immune homeostasis. Concurrently, immunomodulatory strategies advance toward multi-target interventions, with combination therapies targeting Th2/Th17 pathways and specific immune cell functions to achieve multidimensional inflammatory cascade inhibition.

## 7 Conclusions

AD pathogenesis centers on an interconnected pathological triad:microbiome dysbiosis, barrier dysfunction, and Th2 immune deviation. Therapeutic strategies targeting skin microbiota restoration represent promising disease-modifying interventions for AD.Despite the enormous potential of microbiota-based AD treatments, the treatment of ecological disorders should be integrated with standard skincare practices, as AD is a complex dermatological condition that requires a multifaceted approach combining a variety of skincare modalities and therapeutic approaches for effective management. Despite promising evidence from clinical studies and early phage therapy trials for microbiota-targeted AD treatments, key mechanistic insights remain elusive, and critical translational questions require resolution for optimal clinical implementation. Developing an integrated host-microbiome model is imperative for advancing AD management, as skin microbiota dynamics are shaped by complex interactions between host physiology and environmental exposures. Current research on skin microbiota- AD interactions primarily employs *in vitro* and murine models, with select therapeutic candidates advancing to early-phase clinical trials (Phase I/II). Future research must leverage multi-omics approaches to resolve spatiotemporal dynamics of host-microbe interactions and develop personalized therapies concurrently targeting: microbiome remodeling, Immune recalibration and barrier restoration. These precision strategies are poised for near-term clinical translation in AD management.
